# Rhesus Cytomegalovirus-Specific CD8^+^ Cytotoxic T Lymphocytes Do Not Become Functionally Exhausted in Chronic SIVmac239 Infection

**DOI:** 10.3389/fimmu.2020.01960

**Published:** 2020-08-12

**Authors:** Brandon C. Rosen, Nuria Pedreño-Lopez, Michael J. Ricciardi, Jason S. Reed, Jonah B. Sacha, Eva G. Rakasz, David I. Watkins

**Affiliations:** ^1^Medical Scientist Training Program, University of Miami Miller School of Medicine, Miami, FL, United States; ^2^Department of Pathology, University of Miami Miller School of Medicine, Miami, FL, United States; ^3^Department of Pathology, George Washington University School of Medicine, Washington, DC, United States; ^4^Vaccine and Gene Therapy Institute, Oregon Health & Science University, Beaverton, OR, United States; ^5^Oregon National Primate Research Center, Oregon Health & Science University, Beaverton, OR, United States; ^6^Wisconsin National Primate Research Center, University of Wisconsin-Madison, Madison, WI, United States

**Keywords:** simian immunodeficiency virus, cytotoxic T lymphocytes, cytomegalovirus, rhesus macaques, human immunodeficiency virus

## Abstract

CD8^+^ cytotoxic T lymphocytes (CTLs) exert potent antiviral activity after HIV/SIV infection. However, efforts to harness the antiviral efficacy of CTLs for HIV/SIV prophylaxis and therapy have been severely hindered by two major problems: viral escape and exhaustion. By contrast, CTLs directed against human cytomegalovirus (HCMV), a ubiquitous chronic herpesvirus, seldom select for escape mutations and remain functional and refractory to exhaustion during chronic HCMV and HIV infection. Recently, attempts have been made to retarget HCMV-specific CTLs for cancer immunotherapy. We speculate that such a strategy may also be beneficial in the context of HIV/SIV infection, facilitating CTL-mediated control of HIV/SIV replication. As a preliminary assessment of the validity of this approach, we investigated the phenotypes and functionality of rhesus CMV (RhCMV)-specific CTLs in SIVmac239-infected Indian rhesus macaques (RMs), a crucial HIV animal model system. We recently identified two immunodominant, *Mamu-A^∗^02*-restricted CTL epitopes derived from RhCMV proteins and sought to evaluate the phenotypic and functional characteristics of these CTL populations in chronic SIVmac239 infection. We analyzed and directly compared RhCMV- and SIVmac239-specific CTLs during SIVmac239 infection in a cohort of *Mamu-A^∗^01*^+^ and *Mamu-A^∗^02*^+^ RMs. CTL populations specific for at least one of the RhCMV-derived CTL epitopes were detected in ten of eleven *Mamu-A^∗^02*^+^ animals tested, and both populations were detected in five of these animals. Neither RhCMV-specific CTL population exhibited significant changes in frequency, memory phenotype, granzyme B expression, exhaustion marker (PD-1 and CTLA-4) expression, or polyfunctionality between pre- and chronic SIVmac239 infection timepoints. In chronic SIVmac239 infection, RhCMV-specific CTLs exhibited higher levels of granzyme B expression and polyfunctionality, and lower levels of exhaustion marker expression, than SIVmac239-specific CTLs. Additionally, compared to SIVmac239-specific CTLs, greater proportions of RhCMV-specific CTLs were of the terminally differentiated effector memory phenotype (CD28^–^ CCR7^–^) during chronic SIVmac239 infection. These results suggest that, in contrast to SIVmac239-specific CTLs, RhCMV-specific CTLs maintain their phenotypes and cytolytic effector functions during chronic SIVmac239 infection, and that retargeting RhCMV-specific CTLs might be a promising SIV immunotherapeutic strategy.

## Introduction

Development of an efficacious vaccine and a sterilizing cure remain the two principal objectives of the global HIV/AIDS eradication effort. Harnessing the potent antiviral activity of CD8^+^ cytotoxic T lymphocytes (CTLs) is an attractive approach for both prophylactic and therapeutic strategies. CTLs exhibit remarkable potency and are largely responsible for controlling peak viremia to setpoint levels during acute infection (1–4). Paradoxically, the powerful antiviral activity of CTLs results in selection for viral escape mutants, and the subsequent emergence and proliferation of virus subpopulations which are no longer sensitive to CTL-mediated killing (5–7). While viral escape from some CTL populations occurs during acute infection, escape from others can take years (7–9). This latter group of CTL populations is uniquely susceptible to CTL exhaustion, in which chronic, repetitive antigen stimulation results in generalized CTL dysfunction (10–12). Thus, it is likely that the efficacy of nearly all HIV-specific CTL populations is impaired by CTL escape, CTL exhaustion, or both. These phenomena are exquisitely recapitulated in SIVmac239 infection of Indian rhesus macaques (RMs) (7, 8, 13) a HIV/AIDS model system critical for the development, testing, and clinical translation of novel prophylaxis and cure strategies.

Previous studies have suggested that some CTLs directed against other chronic viruses do not become exhausted, even after years of antigenic stimulation. In particular, CTLs specific for the chronic herpesvirus human cytomegalovirus (HCMV) increase in frequency as an individual ages, culminating in high frequencies of HCMV-specific CTLs in the elderly (14, 15). HCMV-specific CTLs thus retain a long-term capability to proliferate and execute effector functions in response to antigenic stimulation (14, 16, 17). Even in the context of chronic HIV infection, HCMV-specific CTLs appear to maintain a late-differentiated effector memory phenotype and polyfunctional effector function profile (12). In contrast, HIV- and SIV-specific CTLs exhibit impaired proliferative capacity, increased susceptibility to activation-induced apoptosis, and elevated exhaustion marker expression during chronic infection (10–13). However, it should be noted that in the context of severe AIDS (generally <50 CD4^+^ T cells/μL in humans), HCMV-specific CTLs can become dysfunctional, presumably due to inadequate CD4^+^ T cell help (18). This often results in HCMV-induced disease (e.g., retinitis, colitis, and esophagitis). Similarly, CMV-induced disease is observed in chronically SIV-infected RMs with low CD4^+^ T cell counts and dysfunctional CMV-specific CTLs (19). Nevertheless, in both humans and non-human primates, CMV-specific CTLs appear to retain functionality until end-stage AIDS and would likely remain efficacious throughout most of chronic HIV/SIV infection, even in the absence of antiretroviral therapy (ART).

Retargeting CMV-specific CTLs has been previously investigated as a cancer immunotherapeutic strategy. Using fusion proteins comprised of peptide-MHC I (pMHCI) and monoclonal antibody (mAb) domains, Schmittnaegel et al. demonstrated that HCMV-specific CTLs could be redirected to kill tumor cells using these pMHCI-mAb fusion proteins, both *in vitro* and in a murine xenograft model (20, 21). Other classes of T cell retargeting molecules, including bispecific T cell engagers (BiTEs) and dual-affinity retargeting molecules (DARTs), have demonstrated some efficacy in the context of HIV/SIV infection, using anti-Envelope domains to target infected cells (22, 23). Although the BiTEs and DARTs tested in these studies did not recruit specific T cell populations, due to their promiscuous anti-CD3 domains, it is conceivable that BiTEs and DARTs that recognize T cell receptors of a given specificity (i.e., for an MHC I molecule loaded with a CMV-derived peptide) could be generated in the future. Nevertheless, to our knowledge, retargeting endogenous antigen-specific CTL populations to kill HIV/SIV-infected cells has never been attempted. Thus, we sought to identify antigen-specific CTL populations that could be suitable for *in vivo* retargeting in the SIVmac239 model of HIV infection in RMs.

While the phenotypic and functional characteristics of HCMV-specific CTLs in chronic HIV infection have been studied, those of rhesus CMV (RhCMV)-specific CTLs in chronic SIVmac239 infection have not. Although nearly 100% of adult RMs housed at primate centers are RhCMV-infected (24), until recently, no minimal optimal CTL epitopes derived from RhCMV proteins had been defined. We identified two immunodominant RhCMV-derived CTL epitopes restricted for Mamu-A^∗^02, a high-frequency RM major histocompatibility class I (MHC I) molecule (unpublished data; (25)). We decided to further characterize these two RhCMV-specific CTL populations in the context of SIVmac239 infection. We hypothesized that RhCMV-specific CTLs would remain functional and refractory to exhaustion throughout SIVmac239 infection, similarly to HCMV-specific CTLs during HIV infection. Using our historical cohort of unvaccinated, SIVmac239-infected RMs, we analyzed and compared the phenotypic and functional properties of RhCMV- and SIVmac239-specific CTLs during SIVmac239 infection. In addition to our two Mamu-A^∗^02-restricted RhCMV-specific CTL populations, we studied four well-characterized SIVmac239-specific CTL populations: those specific for the Mamu-A^∗^01-restricted epitopes Tat SL8 and Gag CM9 (7, 26, 27), and those specific for the Mamu-A^∗^02-restricted epitopes Nef YY9 and Gag GY9 (28, 29). We found that the phenotypic and functional characteristics of RhCMV-specific CTLs did not change between pre- and chronic SIVmac239 infection timepoints, and that RhCMV-specific CTLs were generally better-suited for execution of effector functions than SIVmac239-specific CTLs during chronic SIVmac239 infection. Here we discuss the implications of these findings for the development of CTL-based immunotherapeutic strategies for HIV/SIV.

## Materials and Methods

### Research Animals and Ethics Statement

The 18 Indian rhesus macaques (RMs; *Macaca mulatta*) whose peripheral blood mononuclear cells (PBMCs) were analyzed in this study served as unvaccinated controls for our previous vaccination studies (Table 1) (30–33). All animals were housed at the Wisconsin National Primate Research Center (WNPRC) and cared for in compliance with the guidelines of the Weatherall report (34) and the National Research Council’s *Guide for the Use and Care of Laboratory Animals* (35). All procedures were performed in accordance with protocols approved by the University of Wisconsin Graduate School Animal Care and Use Committee. Detailed descriptions of caretaking procedures (i.e., housing, feeding, environmental enrichment, and medical care) and efforts to minimize animal suffering (i.e., anesthesia and euthanasia) can be found in the “Research Animals and Ethics Statement” sections in the Materials and Methods of our previously published studies (30–33). MHC class I genotyping was conducted by the WNPRC.

**TABLE 1:** Animal characteristics.

Animal ID	MHC Class I Genotype	Age (yrs.)^*a*^	Sex	Reference^*b*^
rh2306	Mamu-A*01, Mamu-A*02	8.1	M	30
rh2313	Mamu-A*01	11.8	M	31
r02108	Mamu-A*01	8.4	M	30
r03116	Mamu-A*01	7.4	M	30
r03141	Mamu-A*01	7.3	M	30
r13086	Mamu-A*01	5.6	M	
r10055	Mamu-A*01, Mamu-B*08	4.7	M	33
r11063	Mamu-A*01, Mamu-B*08	3.7	F	33
r02076	Mamu-A*02	8.6	F	30
r03103	Mamu-A*02	7.5	M	30
r03111	Mamu-A*02	7.5	F	30
r04156	Mamu-A*02	10.6	F	31
r08007	Mamu-A*02	11.1	M	
r09028	Mamu-A*02	8.0	M	
r11075	Mamu-A*02	5.7	F	
r11083	Mamu-A*02	7.6	F	
r00008	Mamu-A*02, Mamu-B*08	15.2	F	33
r10018	Mamu-A*02, Mamu-B*17	5.1	M	32

*^*a*^Animal age at time of infecting SIVmac239 challenge. ^*b*^Reference for published vaccination study in which animal served as unvaccinated control. Animals lacking references were unvaccinated controls for studies which have not yet been published.*

### SIVmac239 Challenges and Viral Load Quantification

**FIGURE 1:**
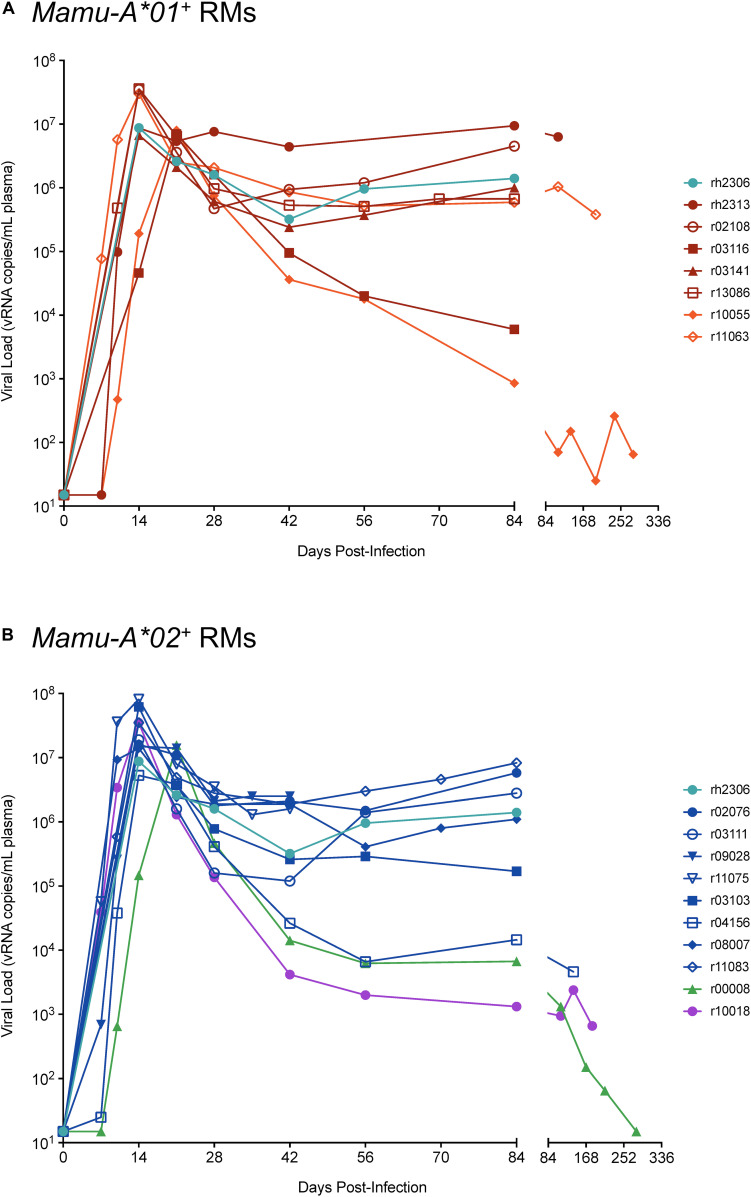
SIVmac239 viral loads for rhesus macaques in this study. **(A)** Mamu-A*01^+^ RMs. **(B)** Mamu-A*02^+^ RMs. Viral loads for rh2306 are shown in both graphs, since this animal was Mamu-A*01^+^ Mamu-A*02^+^.

As described in our previous studies, RMs were subjected to repeated intrarectal challenges with SIVmac239, once every 2 weeks (30–33). Depending on the study, each challenge dose contained either 4.8 × 10^5^ (31–33) or 6.52 × 10^6^ viral RNA (vRNA) copies (30). Plasma viral loads were quantified at 7 and 10 days after each challenge, and animals were no longer challenged if a detectable viral load measurement (>15 vRNA copies/mL plasma) was obtained. In one of our studies, animals were subjected to escalating dose challenges if they remained uninfected after the first eight challenges (30). For all other studies, the same dose was used for all challenges. Viral loads were determined by extracting total RNA from EDTA-anticoagulated plasma and using this RNA as template for a two-step reverse-transcription PCR with Gag-specific primers and a Gag-specific fluorescent probe, as previously described (30–33, 36). Viral loads for each animal are shown in Figure 1.

### Study Design and Animal Population

We conducted a retrospective analysis of RhCMV- and SIVmac239-specific CTL populations in our historical cohort of unvaccinated RMs. Animal inclusion criteria were as follows: (1) no history of vaccination against SIVmac239, (2) a MHC class I haplotype including the *Mamu-A^∗^01* allele, the *Mamu-A^∗^02* allele, or both, and (3) the presence of cryopreserved PBMC samples at an acute SIVmac239 infection timepoint, a chronic SIVmac239 infection timepoint, or both. Acute infection timepoints were defined as those 35–70 days (5–10 weeks) after the infecting challenge. Chronic infection timepoints were defined as those >70 days (>10 weeks) after the infecting challenge. PBMC timepoints for each animal are listed in Table 2. Despite the absence of any defined *Mamu-A^∗^01*-restricted, RhCMV-derived CTL epitopes, we chose to include *Mamu-A^∗^01*^+^ animals in our study, since *Mamu-A^∗^01*-restricted CTLs specific for SIVmac239-derived peptides (i.e., Tat SL8 and Gag CM9) are some of the best-characterized SIVmac239-specific CTL populations (7, 13, 26, 27).

**TABLE 2:** Timepoints for PBMC analysis.

Animal ID	Pre-infection (days)^*a*^	Acute phase (days)^*b*^	Chronic phase (days)^*c*^
rh2306	0	65	141
rh2313		46	165
r02108		57	122
r03116			85
r03141		63	
r13086		60	102
r10055		60	214
r11063			138
r02076	0	56	183
r03103	0	64	92
r03111	15	62	247
r04156	84		146
r08007	87	60	78
r09028	17	54	
r11075	83	37	
r11083	60	60	162
r00008	81		186
r10018	58	55	175

Mean ± Standard Deviation (days)	**48.5 ± 36.6**	**57.1 ± 7.57**	**149.1 ± 48.5**

^*a*^Days before infecting SIVmac239 challenge. Pre-infection timepoints were not analyzed for Mamu-A*01^+^ animals since, to date, no Mamu-A*01-restricted, RhCMV-derived CD8^+^ CTL epitopes have been identified. ^*b*^Acute phase was defined as 5–10 weeks (35–70 days) post-infecting SIVmac239 challenge. ^*c*^Chronic phase was defined as >10 weeks (>70 days) post-infecting SIVmac239 challenge.

### PBMC Isolation and Cryopreservation

PBMCs were isolated from EDTA-anticoagulated blood by Ficoll-Paque Plus (GE Healthcare) density centrifugation and washed once in R10 medium [RPMI 1640 with GlutaMAX (Gibco), 10% heat-inactivated FBS (Gibco), antibacterial/antimycotic (Lonza)]. In some cases, red blood cells were lysed by resuspending PBMCs in ACK lysing buffer (Lonza or Gibco) and incubating for 5 min at room temperature. PBMCs were washed again in R10 medium (if ACK-treated), quantified, then pelleted by centrifugation and resuspended in cryopreservation solution (90% FBS, 10% DMSO; or 67.5% RPMI 1640, 22.5% FBS, 10% DMSO) at 4°C. PBMCs were cooled to −80°C, then transferred to liquid nitrogen freezers for long-term storage. For the immunophenotyping assays in this study (described below), cryopreserved PBMCs were thawed in a 37°C water bath, washed twice in R10 medium, and re-quantified. Approximately 1.5 × 10^6^ PBMCs were used per test.

### Peptide-MHC I (pMHCI) Tetramer Staining

PBMCs were stained with the appropriate BV421-conjugated pMHCI tetramers (NIH Tetramer Core Facility at Emory University, Atlanta, GA, United States) for 30 min at room temperature. PBMCs were then stained for an additional 30 min with a cocktail containing the following fluorophore-conjugated monoclonal antibodies (mAbs) and amine-reactive viability dye: anti-CCR7 FITC (clone 150503, BD Biosciences), anti-CD8a BV711 (clone RPA-T8, BioLegend), anti-CD28 BV785 (clone CD28.2, BioLegend), anti-CD14 APC (clone M5E2, BioLegend), anti-CD20 APC (clone 2H7, BioLegend), anti-CD159a APC (clone Z199, Beckman Coulter), anti-PD-1 PE-Cy7 (clone EH12.2H7, BioLegend), and Far Red live/dead dye (LIVE/DEAD fixable Far Red dead cell stain kit, Invitrogen). Cells were washed, then fixed and permeabilized using Cytofix/Cytoperm (BD Biosciences) and Perm/Wash Buffer (BD Biosciences), respectively. Cells were stained intracellularly with anti-CD3 PerCP-Cy5.5 (clone SP34-2, BD Biosciences), anti-granzyme B Alexa Fluor 700 (clone GB11, BD Biosciences), and anti-CTLA-4 PE-CF594 (clone BNI3, BD Biosciences). Following a final wash in Perm/Wash Buffer, samples were acquired on a special-order BD LSR II flow cytometer. Data was analyzed using FlowJo software (version 10.4); gating strategy is shown in Supplementary Figure 1. To assess the extent of SIVmac239-induced disease progression in our RMs, we calculated approximate CD4:CD8 T cell ratios using the aforementioned pMHCI tetramer staining data from resting PBMCs. CD4:CD8 T cell ratios were calculated using the following formula: (100 – % of CD8^+^ T cells among live CD3^+^ CD14^–^ CD20^–^ CD159a^–^ lymphocytes)/(% of CD8^+^ T cells among live CD3^+^ CD14^–^ CD20^–^ CD159a^–^ lymphocytes). This data is depicted in Supplementary Figure 2.

### CD107a Degranulation Assay With Intracellular Cytokine Staining (CD107a/ICS)

Peripheral blood mononuclear cells in R10 medium (1.5 × 10^6^ per tube) were incubated with anti-CD28 and anti-CD49d costimulatory mAbs (BD Biosciences, clones L293 and 9F10, respectively), anti-CD107a PE (clone H4A3, BioLegend), and minimal optimal peptide (final concentration of 2 μM) for 1 h at 37°C. Following addition of brefeldin A (BioLegend) and GolgiStop (BD Biosciences), samples were incubated for an additional 5 h at 37°C. Cells were washed once, then stained with the following cocktail of fluorophore-conjugated mAbs and amine-reactive viability dye: anti-CD8a BV785 (clone RPA-T8, BioLegend), anti-CD14 APC (clone M5E2, BioLegend), anti-CD20 APC (clone 2H7, BioLegend), anti-CD159a APC (clone Z199, Beckman Coulter), anti-PD-1 PE-Cy7 (clone EH12.2H7, BioLegend), and Far Red live/dead dye (LIVE/DEAD fixable Far Red dead cell stain kit, Invitrogen). Following a wash step, cells were fixed and permeabilized using Cytofix/Cytoperm (BD Biosciences) and Perm/Wash Buffer (BD Biosciences), respectively. Cells were then stained intracellularly with anti-CD3 PerCP-Cy5.5 (clone SP34-2, BD Biosciences), anti-TNF-α APC-Cy7 (clone MAb11, BioLegend), anti-IFN-γ BV421 (clone B27, BioLegend), and anti-CTLA-4 PE-CF594 (clone BNI3, BD Biosciences). Cells were washed once more in Perm/Wash Buffer, and samples were acquired on a special-order BD LSR II flow cytometer. Data was analyzed with FlowJo software (version 10.4); gating strategy is shown in Supplementary Figure 3. To evaluate T cell functionality, we created the following gates: CD107a alone (degranulation), a Boolean *or* gate to quantify overall response frequencies (CD107a *or* IFN-γ *or* TNF-α), and a Boolean *and* gate to assess polyfunctionality (CD107a *and* IFN-γ *and* TNF-α). For each of the above gates, we subtracted the corresponding frequency for the unstimulated negative control (Microsoft Excel). We also analyzed exhaustion marker (PD-1 and CTLA-4) expression in the Boolean *or* gate subpopulation. For degranulation, polyfunctionality, and exhaustion marker analyses, the Boolean *or* gate frequency for a stimulated sample had to be least two times greater than the *or* gate frequency for the unstimulated negative control to be included in our comparisons of multiple animals.

### Statistical Analyses

Comparisons of CTL populations were performed using Welch’s *t*-test (unpaired two-sample *t*-test assuming unequal variances; Microsoft Excel). Associations between CTL phenotypic characteristics and setpoint SIVmac239 viral loads were evaluated by Pearson correlation analysis (GraphPad Prism). *p*-values < 0.05 were considered statistically significant and are reported in tables, figures, or figure legends.

## Results

### Clinical Parameters of SIVmac239 Infection in Unvaccinated RMs

As a preliminary investigation into the suitability of RhCMV-specific CTLs for retargeting-based immunotherapeutic strategies, we conducted a retrospective analysis of 18 unvaccinated, SIVmac239-infected RMs to analyze and compare the phenotypic characteristics and effector functions of RhCMV- and SIVmac239-specific CTLs during SIVmac239 infection (Table 1). Five of the 18 RMs controlled SIVmac239 replication to below 10,000 vRNA copies/mL plasma during chronic infection (Figure 1). Three of the five controllers (r10055, r00008, and r10018) expressed one of the elite control-associated RM MHC I alleles, *Mamu-B^∗^08* or *Mamu-B^∗^17* (37, 38). However, one of three *Mamu-B^∗^08^+^* RMs (r11063) did not control SIVmac239 replication, with setpoint viral loads well above 10^5^ vRNA copies/mL plasma. The extent of SIVmac239-induced immunosuppression was monitored by flow cytometry-based computation of the CD4:CD8 T cell ratio in resting PBMCs of each animal. Average CD4:CD8 T cell ratios decreased from 1.64 pre-SIVmac239 infection to 1.14 in acute infection, and to 0.81 in chronic infection (Supplementary Figure 2).

### Frequencies of RhCMV-Specific CTLs Do Not Change During SIVmac239 Infection

**FIGURE 2:**
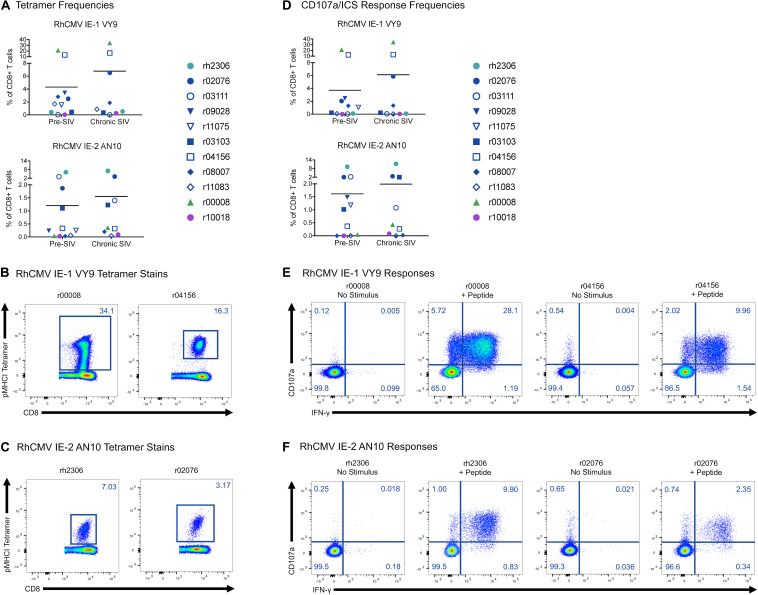
Frequencies of RhCMV-specific CD8^+^ T cells before and during SIVmac239 infection. PBMCs from SIVmac239-infected Mamu-A*02^+^ animals were stained with **(A–C)** the indicated pMHCI tetramers or **(D–F)** stimulated with the corresponding minimal optimal peptides in a CD107a degranulation assay with ICS. Graphs depict the frequencies of antigen-specific CD8^+^ T cells among all CD8^+^ T cells (defined as live CD3^+^ CD8^+^ CD14^–^ CD20^–^ CD159a^–^ lymphocytes). Flow cytometry plots depict all CD8^+^ T cells for the animals with the highest frequencies of RhCMV-specific CD8^+^ T cells in chronic SIVmac239 infection by tetramer staining **(B,C)** or CD107a degranulation assay with ICS **(E,F)**. None of the differences in tetramer or CD107a/ICS frequencies between pre- and chronic infection timepoints were statistically significant (all *p* > 0.05 by Welch’s *t*-test).

To determine whether RhCMV-specific CTLs undergo changes in frequency, phenotype, or effector function repertoire during SIVmac239 infection, we studied RhCMV-specific CTL populations in the PBMCs of eleven *Mamu-A^∗^02*^+^ RMs prior to SIVmac239 infection and during chronic SIVmac239 infection (defined as >10 weeks after the infecting SIVmac239 challenge; Table 2). We focused on two recently identified CTL populations specific for the RhCMV-derived peptides IE-1 VY9 (25) and IE-2 AN10 (unpublished data). We identified these antigen-specific CTL populations by two methods: pMHCI tetramer staining of resting PBMCs, and CD107a degranulation assay with ICS (CD107a/ICS) in which PBMCs were stimulated with the minimal optimal peptides IE-1 VY9 or IE-2 AN10. At least one of the two RhCMV-specific CTL populations was present in ten of eleven RMs tested (Figure 2). Nine RMs had detectable IE-1 VY9-specific populations, while six had detectable IE-2 AN10 populations. Both CTL populations were present in five of the eleven animals. The tetramer staining and CD107a/ICS analyses yielded relatively consistent results, suggesting that most circulating RhCMV-specific CTLs are responsive to stimulation with their cognate peptide. Frequencies of RhCMV-specific CTLs did not differ significantly between pre-SIVmac239 infection and chronic SIVmac239 infection timepoints (all *p*-values > 0.05, Welch’s *t*-test). On average, RhCMV IE-1 VY9-specific CTLs comprised 4–7% of circulating CD8^+^ T cells, while RhCMV IE-2 AN10-specific CTLs accounted for 1–2% of circulating CD8^+^ T cells. Remarkably, tetramer frequencies and CD107a/ICS response frequencies for RhCMV IE-1 VY9 exceeded 10% of circulating CD8^+^ T cells in two animals, and RhCMV IE-1 VY9-specific CTLs accounted for 34% of circulating CD8^+^ T cells in one animal (r00008).

### Phenotypic Characteristics and Effector Function Profiles of RhCMV-Specific CTLs Do Not Change During SIVmac239 Infection

**FIGURE 3:**
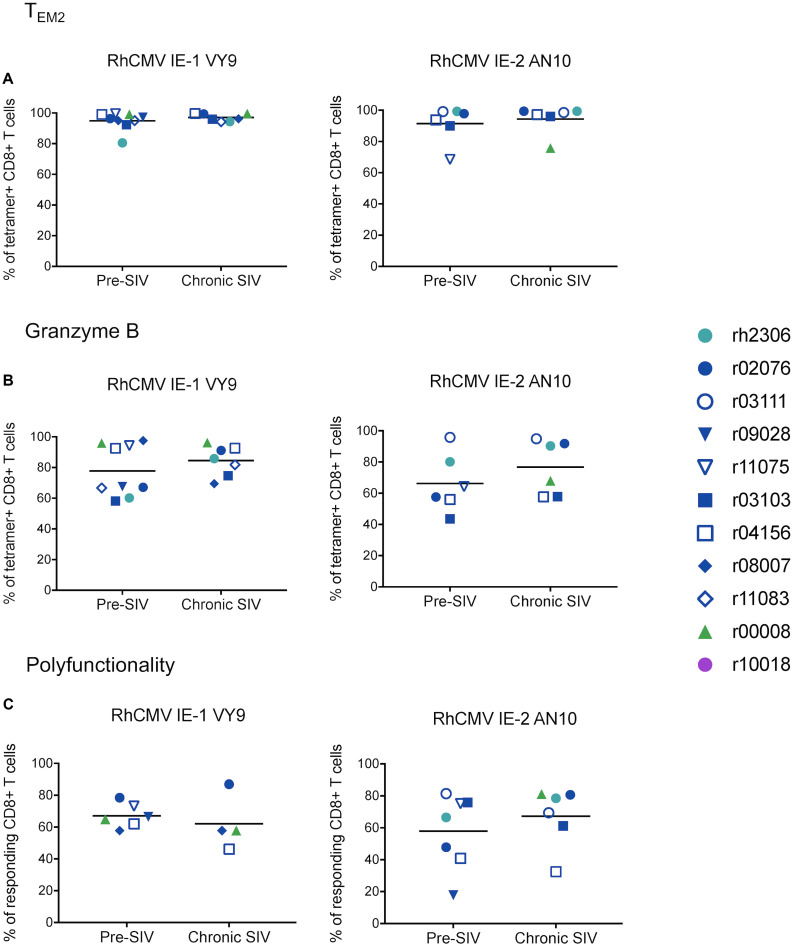
Phenotypic and functional characteristics of RhCMV-specific CD8^+^ T cells before and during SIVmac239 infection. Unstimulated PBMCs were stained with pMHCI tetramer and fluorophore-conjugated mAbs to determine the frequencies of **(A)** T_EM2_ (CD28^–^ CCR7^–^) and **(B)** granzyme B^+^ cells among tetramer^+^ CD8^+^ T cells (defined as live CD3^+^ CD8^+^ tetramer^+^ CD14^–^ CD20^–^ CD159a^–^ lymphocytes). **(C)** CTL polyfunctionality was evaluated by CD107a/ICS assay, in which PBMCs were stimulated with the corresponding minimal optimal peptides. A responding CD8^+^ T cell (a live CD3^+^ CD8^+^ CD14^–^ CD20^–^ CD159a^–^ lymphocyte) was defined as a cell staining positive for CD107a or IFN-γ or TNF-α. Polyfunctionality was defined as the proportion of responding CD8^+^ T cells staining for all three markers simultaneously (CD107a^+^ IFN-γ^+^ TNF-α^+^). None of the differences between pre- and chronic-phase CD8^+^ T cells in panels **(A–C)** were statistically significant (all *p* > 0.05 by Welch’s *t*-test).

**FIGURE 4:**
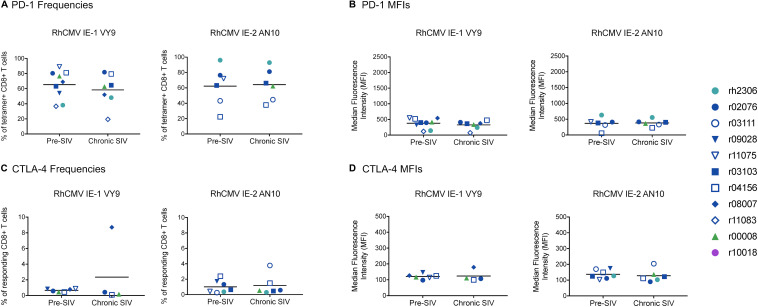
Exhaustion marker expression by RhCMV-specific CD8^+^ T cells before and during SIVmac239 infection. **(A)** Frequencies of PD-1^+^ CTLs and **(B)** PD-1 median fluorescence intensities (MFIs) were determined by pMHCI tetramer staining of unstimulated PBMCs. PD-1 expression was analyzed in tetramer^+^ CD8^+^ T cells (defined as live CD3^+^ CD8^+^ tetramer^+^ CD14^–^ CD20^–^ CD159a^–^ lymphocytes). **(C)** Frequencies of CTLA-4^+^ CTLs and **(D)** CTLA-4 MFIs were evaluated by CD107a/ICS assay, in which PBMCs were stimulated with the corresponding minimal optimal peptides. CTLA-4 expression was analyzed in responding CD8^+^ T cells, which were defined as live CD3^+^ CD8^+^ CD14^–^ CD20^–^ CD159a^–^ lymphocytes staining positive for CD107a or IFN-γ or TNF-α. None of the differences between pre- and chronic-phase CD8^+^ T cells in panels **(A–D)** were statistically significant (all *p* > 0.05 by Welch’s *t*-test).

To determine whether the phenotypic characteristics or effector functionalities of RhCMV-specific CD8^+^ CTLs become altered during SIVmac239 infection, we examined a number of characteristics integral to the functionality and efficacy of a CTL. These features included memory phenotype, exhaustion marker expression, direct markers of cytolytic effector function (granzyme B expression and degranulation upon stimulation), and expression of cytokines upon stimulation. CTLs specific for the RhCMV-derived peptides IE-1 VY9 and IE-2 AN10 were defined by tetramer staining and CD107a/ICS assays, as described above. Memory phenotype, granzyme B expression, and PD-1 expression were evaluated in tetramer^+^ CTLs in resting, unstimulated PBMCs. Both CTL populations were predominantly of the terminally differentiated effector memory (T_EM2_) phenotype, defined as CD28^–^ CCR7^–^ CTLs (39), and the frequencies of T_EM2_ CTLs did not change during SIVmac239 infection (Figure 3A). High frequencies of CTLs specific for both RhCMV epitopes expressed granzyme B, and granzyme B^+^ frequencies remained at similarly high levels during chronic SIVmac239 infection (Figure 3B). Interestingly, the exhaustion marker PD-1 was expressed at relatively high levels by both RhCMV-specific CTL populations before and during SIVmac239 infection, although the frequencies of PD-1^+^ CTLs and PD-1 median fluorescence intensities (MFIs) did not differ significantly between these timepoints (Figures 4A,B). We evaluated CTL effector functions and expression of the exhaustion marker CTLA-4 via CD107a/ICS assays in which PBMCs were stimulated with the RhCMV-derived minimal optimal peptides. In contrast to PD-1, very low frequencies of RhCMV-specific CTLs expressed CTLA-4, even during chronic SIVmac239 infection (Figure 4C). CTLA-4 MFIs for RhCMV-specific CTLs were similarly low (Figure 4D). At both pre- and chronic SIVmac239 infection timepoints, the majority of CTLs responding to either of the RhCMV peptides were polyfunctional, which was defined as simultaneous expression of all three effector function markers (CD107a^+^ IFN-γ^+^ TNF-α^+^) (Figure 3C). In summary, we observed no significant differences in the phenotypic characteristics or effector function profiles of RhCMV IE-1 VY9- or RhCMV IE-2 AN10-specific CTLs between pre- and chronic SIVmac239 infection timepoints.

### Most Circulating SIVmac239-Specific CTLs Do Not Undergo Significant Changes in Frequency, Phenotype, or Effector Functionalities Between Acute and Chronic Infection Timepoints

HIV/SIV-specific CTLs are often characterized as “dysfunctional” or “exhausted” during chronic infection (10–13). To further evaluate our hypothesis that RhCMV-specific CTLs are refractory to the generalized immune dysfunction that occurs during SIVmac239 infection, we directly compared our two RhCMV-specific CTL populations with four well-characterized, immunodominant SIVmac239-specific CTL populations during chronic SIVmac239 infection. These included Mamu-A^∗^01-restricted populations specific for Tat SL8 and Gag CM9 (7, 26, 27), as well as Mamu-A^∗^02-restricted populations specific for Nef YY9 and Gag GY9 (28, 29). We analyzed the PBMCs of seven *Mamu-A^∗^01*^+^ RMs, ten *Mamu-A^∗^02*^+^ RMs, and one *Mamu-A^∗^01*^+^ *Mamu-A^∗^02*^+^ RM by tetramer staining and CD107a/ICS assays at acute and chronic SIVmac239 infection timepoints (Tables 1, 2). Interestingly, we observed no statistically significant differences in the frequencies of CTLs specific for any of the four SIVmac239-derived epitopes between acute and chronic infection (Supplementary Figure 4). Notably, the frequencies of Tat SL8-specific CTLs in chronic infection were two- to four-fold lower than in acute infection, but these differences were not statistically significant (*p* = 0.2204 for tetramer frequencies, *p* = 0.3392 for CD107a/ICS response frequencies). Furthermore, we observed no statistically significant differences in the frequencies of T_EM2_, granzyme B^+^, polyfunctional, PD-1^+^, or CTLA-4^+^ SIVmac239-specific CTLs between acute and chronic infection timepoints (Supplementary Figures 5, 6). The average granzyme B^+^ frequencies of Tat SL8-specific CTLs did decrease twofold from acute to chronic infection, although this reduction was not statistically significant (*p* = 0.2486).

### RhCMV-Specific CTLs Exhibit Greater Functionality Than SIVmac239-Specific CTLs During Chronic SIVmac239 Infection

**FIGURE 5:**
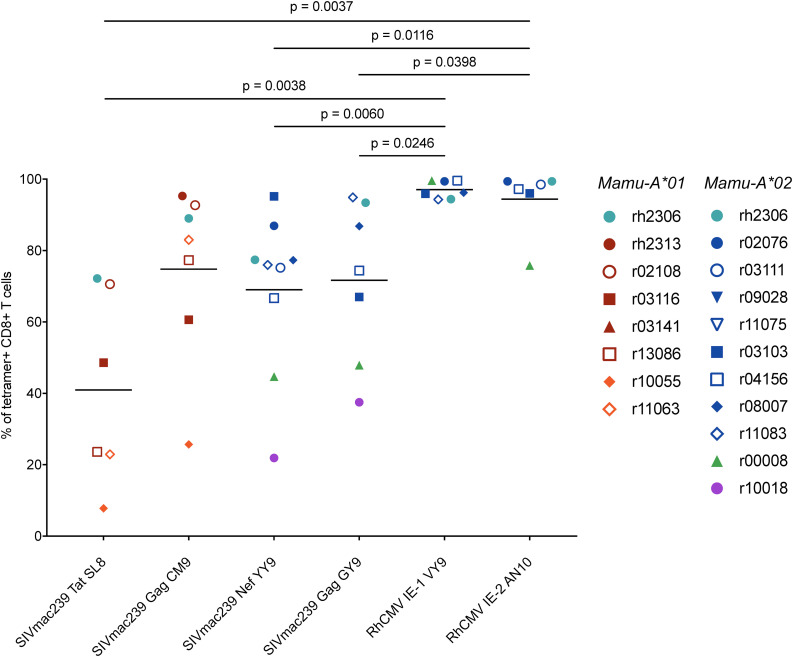
Frequencies of T_EM2_ lymphocytes among RhCMV- and SIVmac239-specific CD8^+^ T cells during chronic SIVmac239 infection. Graphs depict the frequencies of T_EM2_ (CD28^–^ CCR7^–^) cells among tetramer^+^ CD8^+^ T cells (defined as live CD3^+^ CD8^+^ tetramer^+^ CD14^–^ CD20^–^ CD159a^–^ lymphocytes). Statistical analysis was conducted using Welch’s *t*-test. Comparison of T_EM2_ frequencies between Tat SL8- and Gag CM9-specific CD8^+^ T cells yielded a *p*-value of 0.0407 (Welch’s *t*-test; not shown in figure). All other comparisons of RhCMV- and/or SIVmac239-specific CD8^+^ T cells without *p*-values listed in figure yielded non-significant *p*-values (*p* > 0.05).

**FIGURE 6:**
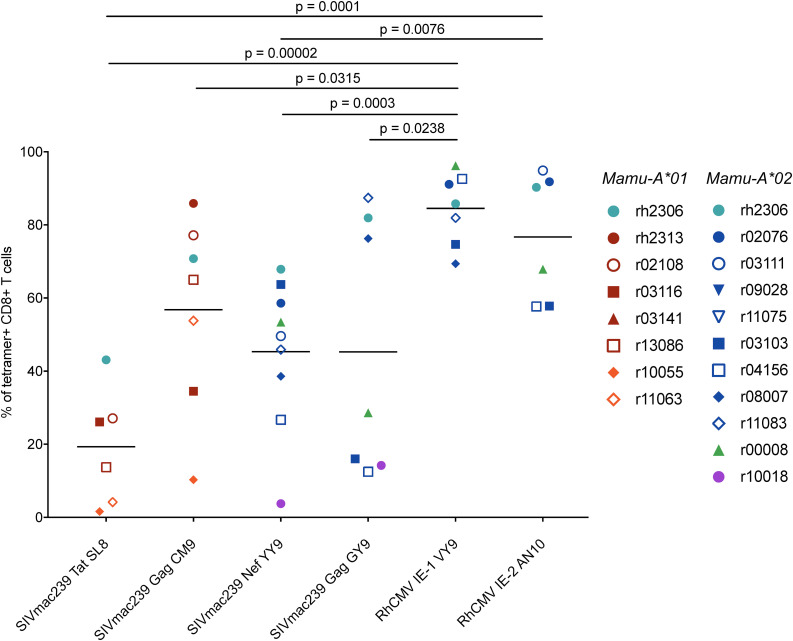
Granzyme B expression in RhCMV- and SIVmac239-specific CD8^+^ T cells during chronic SIVmac239 infection. Graph depicts the frequencies of granzyme B^+^ cells among tetramer^+^ CD8^+^ T cells (defined as live CD3^+^ CD8^+^ tetramer^+^ CD14^–^ CD20^–^ CD159a^–^ lymphocytes). Statistical analysis was conducted using Welch’s *t*-test. The following comparisons of granzyme B frequencies in SIVmac239-specific CD8^+^ T cell populations also yielded statistically significant *p*-values: Tat SL8- and Gag CM9-specific CD8^+^ T cells (*p* = 0.0103); Tat SL8- and Nef YY9-specific CD8^+^ T cells (*p* = 0.0150). All other comparisons of RhCMV- and/or SIVmac239-specific CD8^+^ T cells without *p*-values listed in figure yielded non-significant *p*-values (*p* > 0.05).

**FIGURE 7:**
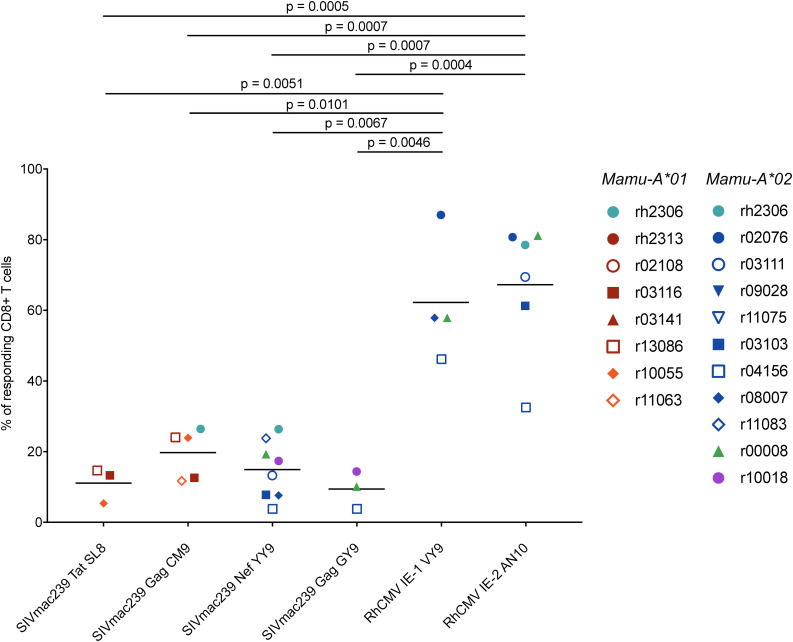
Frequencies of polyfunctional lymphocytes (CD107a^+^ IFN-γ^+^ TNF-α^+^) among RhCMV- and SIVmac239-specific CD8^+^ T cells during chronic SIVmac239 infection. Graph depicts the frequencies of polyfunctional (CD107a^+^ IFN-γ^+^ TNF-α^+^) CD8^+^ T cells responding to minimal optimal peptide in a CD107a degranulation assay with ICS. A responding CD8^+^ T cell was defined as a live CD3^+^ CD8^+^ CD14^–^ CD20^–^ CD159a^–^ lymphocyte staining positive for CD107a or IFN-γ or TNF-α. Statistical significance was evaluated using Welch’s *t*-test. None of the comparisons between SIVmac239-specific CD8^+^ T cell populations yielded statistically significant *p*-values (not shown on graph, all *p* > 0.05).

We then compared the phenotypic and functional characteristics of the four SIVmac239-specific and two RhCMV-specific CTL populations at the chronic SIVmac239 infection timepoint. Both RhCMV-specific CTL populations had significantly higher frequencies of T_EM2_ CTLs than all SIVmac239-specific CTL populations, except for Gag CM9-specific CTLs (Figure 5). Nevertheless, T_EM2_ frequencies for Gag CM9-specific CTLs (mean = 74.8%) were lower than those of RhCMV IE-1 VY9- (mean = 97.1%) and RhCMV IE-2 AN10- (mean = 94.4%) specific CTLs. Both RhCMV-specific CTL populations contained higher frequencies of granzyme B^+^ CTLs than any of the four SIVmac239-specific CTL populations (Figure 6). The differences in granzyme B^+^ frequencies between RhCMV IE-1 VY9- and SIVmac239-specific CTLs were all statistically significant, while the granzyme B^+^ frequencies of RhCMV IE-2 AN10-specific CTLs were significantly higher than those of CTLs specific for Tat SL8 and Nef YY9, but not Gag CM9 and Gag GY9. Most strikingly, frequencies of polyfunctional CTLs were significantly higher in both RhCMV-specific CTL populations than any of the four SIVmac239-specific CTL populations (Figure 7). However, we observed no significant differences in the frequencies of degranulating (CD107a^+^) CTLs among responding RhCMV- and SIVmac239-specific CTLs (Supplementary Figure 7).

### RhCMV-Specific CTLs Express Lower Levels of Exhaustion Markers Than Gag CM9- and Nef YY9-Specific CTLs in Chronic SIVmac239 Infection

**FIGURE 8:**
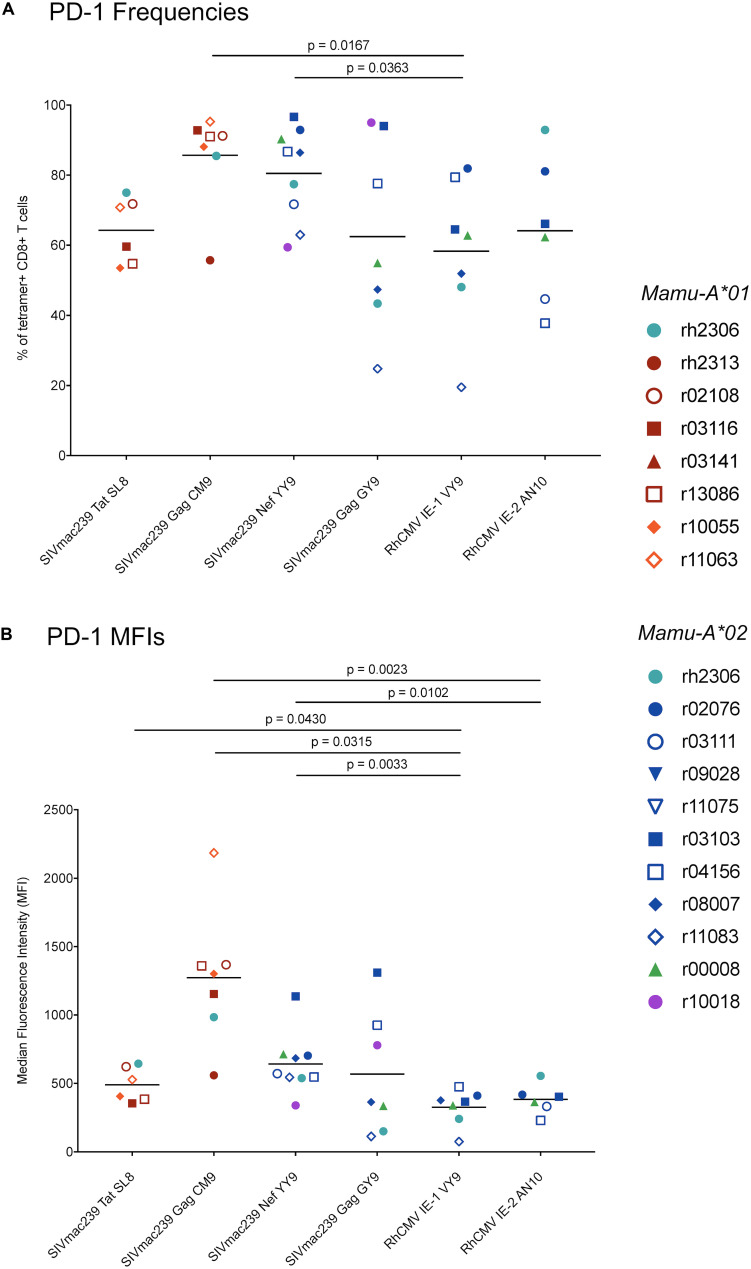
PD-1 expression by RhCMV- and SIVmac239-specific CD8^+^ T cells during chronic SIVmac239 infection. Graph depicts **(A)** frequencies of PD-1^+^ cells among tetramer^+^ CD8^+^ T cells and **(B)** PD-1 median fluorescence intensities (MFIs) for tetramer^+^ CD8^+^ T cells (defined as live CD3^+^ CD8^+^ tetramer^+^ CD14^–^ CD20^–^ CD159a^–^ lymphocytes). Statistical analysis was conducted using Welch’s *t*-test. The following comparisons of PD-1^+^ frequencies between SIVmac239-specific CD8^+^ T cell populations yielded statistically significant *p*-values: Tat SL8- and Gag CM9-specific CD8^+^ T cells (*p* = 0.0066); Tat SL8- and Nef YY9-specific CD8^+^ T cells (*p* = 0.0160). The following comparisons of PD-1 MFIs for SIVmac239-specific CD8^+^ T cells yielded statistically significant *p*-values: Tat SL8- and Gag CM9-specific CD8^+^ T cells (*p* = 0.0048); Gag CM9- and Nef YY9-specific CD8^+^ T cells (*p* = 0.0134); Gag CM9- and Gag GY9-specific CD8^+^ T cells (*p* = 0.0159). All other comparisons of RhCMV- and/or SIVmac239-specific CD8^+^ T cells without *p*-values listed in figure yielded non-significant *p*-values (*p* > 0.05).

**FIGURE 9:**
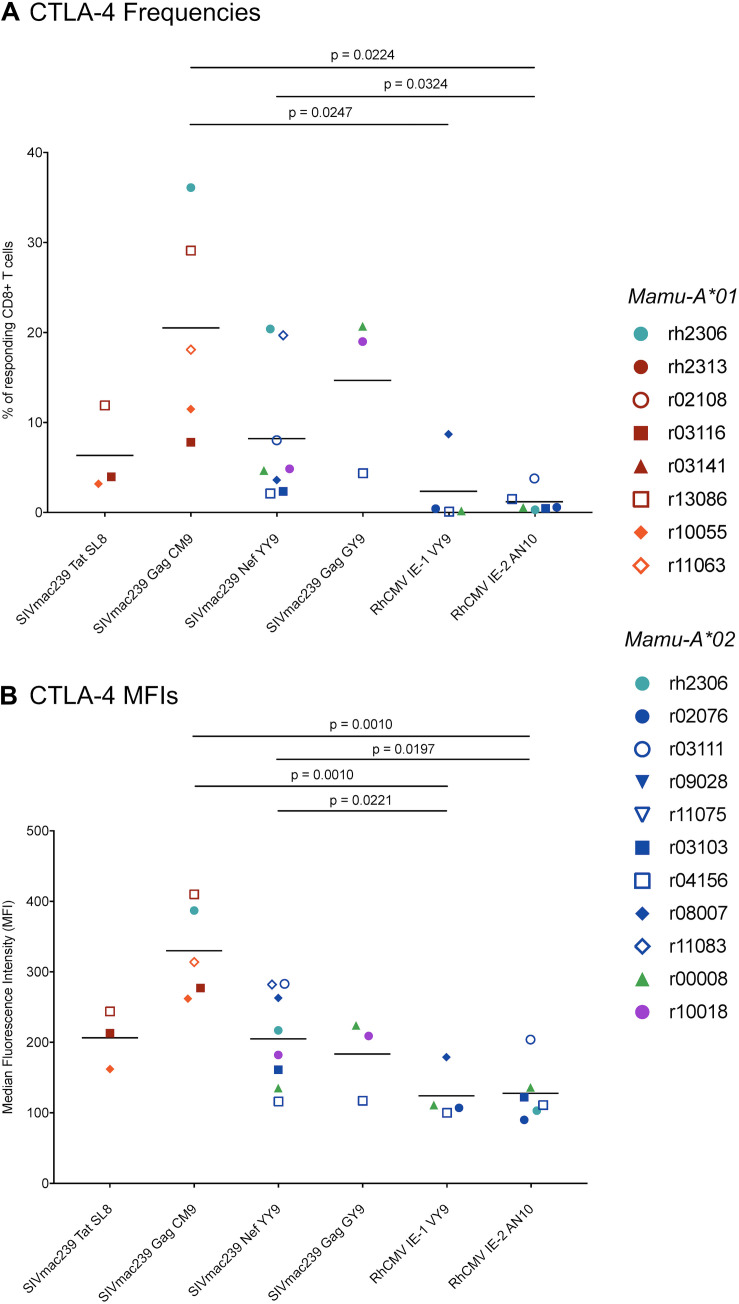
CTLA-4 expression by RhCMV- and SIVmac239-specific CD8^+^ T cells during chronic SIVmac239 infection. Graph depicts **(A)** frequencies of CTLA-4^+^ lymphocytes among CD8^+^ T cells responding to minimal optimal peptide and **(B)** CTLA-4 median fluorescence intensities (MFIs) for responding CD8^+^ T cells in a CD107a/ICS assay. A responding CD8^+^ T cell was defined as a live CD3^+^ CD8^+^ CD14^–^ CD20^–^ CD159a^–^ lymphocyte staining positive for CD107a or IFN-γ or TNF-α. Statistical significance was evaluated using Welch’s *t*-test. None of the comparisons of CTLA-4 frequencies between SIVmac239-specific CD8^+^ T cell populations yielded statistically significant *p*-values (all *p* > 0.05). The following comparisons of CTLA-4 MFIs for SIVmac239-specific CD8^+^ T cells yielded statistically significant *p*-values: Tat SL8- and Gag CM9-specific CD8^+^ T cells (*p* = 0.0172); Gag CM9- and Nef YY9-specific CD8^+^ T cells (*p* = 0.0089); Gag CM9- and Gag GY9-specific CD8^+^ T cells (*p* = 0.0217). All other comparisons of RhCMV- and/or SIVmac239-specific CD8^+^ T cells without *p*-values listed in figure yielded non-significant *p*-values (*p* > 0.05).

To determine whether RhCMV-specific CTLs exhibit features of exhaustion during chronic SIVmac239 infection, we compared the frequencies of CTLs expressing the exhaustion markers PD-1 and CTLA-4 in RhCMV- and SIVmac239-specific CTL populations. On average, both RhCMV-specific CTL populations had lower PD-1^+^ frequencies than two of the SIVmac239-specific CTL populations (Gag CM9 and Nef YY9), and PD-1^+^ frequencies comparable to the other two SIVmac239-specific CTL populations (Tat SL8 and Gag GY9) (Figure 8A). Comparisons of PD-1 MFIs for the RhCMV- and SIVmac239-specific CTL populations yielded similar results, indicating that both RhCMV-specific CTL populations had significantly lower PD-1 MFIs than SIVmac239 Gag CM9- and Nef YY9-specific CTLs (Figure 8B). We found that lower frequencies of RhCMV-specific CTLs expressed CTLA-4 upon stimulation than any of the four SIVmac239-specific CTL populations studied (Figure 9A). Gag CM9-specific CTLs had significantly higher CTLA-4^+^ frequencies than both RhCMV-specific CTL populations, while Nef YY9-specific CTLs had significantly higher CTLA-4^+^ frequencies than RhCMV IE-2 AN10-specific CTLs, but not RhCMV IE-1 VY9-specific CTLs. CTLA-4 MFIs showed a similar trend, with both RhCMV-specific CTL populations exhibiting significantly lower CTLA-4 MFIs than SIVmac239 Gag CM9- and Nef YY9-specific CTLs (Figure 9B).

### Some Phenotypic Characteristics of RhCMV- and SIVmac239-Specific CTLs Correlate With Setpoint SIVmac239 Viral Loads

Five of the 18 RMs in this study controlled chronic-phase SIVmac239 viremia to less than 10,000 vRNA copies/mL plasma. Due to the association between SIV-mediated immunosuppression and increased RhCMV replication, we surmised that associations between chronic-phase SIVmac239 viremia and the phenotypic characteristics of RhCMV-specific CTLs may exist. Additionally, we sought to evaluate potential relationships between phenotypic characteristics of SIVmac239-specific CTLs and viral loads. Pearson correlation analyses revealed multiple statistically significant associations between CTL phenotype and chronic-phase SIVmac239 viremia (Table 3). Setpoint SIVmac239 viral loads were positively correlated with RhCMV IE-1 VY9-specific CTL polyfunctionality and RhCMV IE-2 AN10-specific CTL granzyme B expression. CTLA-4 expression was positively correlated with setpoint viral load for three of the four SIVmac239-specific CTL populations we analyzed. Surprisingly, PD-1 expression by Gag CM9-specific CTLs was negatively correlated with setpoint viral load. When we analyzed all four SIVmac239-specific CTL populations together, we detected positive correlations between T_EM2_ and granzyme B^+^ frequencies, and setpoint viral load.

**TABLE 3:** Correlations between CTL phenotypic parameters and setpoint SIVmac239 viral loads^*a*^.

CTL population	CTL phenotypic parameter	*n*^*b*^	Correlation coefficient (Pearson r)	*p*-value^*c*^
SIVmac239 Tat SL8	CTLA-4^+^ frequency	3	0.9977	0.0429
SIVmac239 Gag CM9	PD-1^+^ frequency	7	−0.9188	0.0035
SIVmac239 Gag CM9	CTLA-4^+^ frequency	5	0.9340	0.0202
SIVmac239 Nef YY9	CTLA-4^+^ frequency	8	0.7307	0.0395
SIVmac239 Nef YY9	CTLA-4 MFI	8	0.7366	0.0371
RhCMV IE-1 VY9	Polyfunctionality	4	0.9606	0.0394
RhCMV IE-2 AN10	Granzyme B^+^ frequency	6	0.8185	0.0464
All SIVmac239	T_EM2_ (CD28^–^ CCR7^–^) frequency	29	0.5092	0.0048
All SIVmac239	Granzyme B^+^ frequency	29	0.5554	0.0018
All RhCMV	Polyfunctionality	10	0.6637	0.0364

*^*a*^Setpoint SIVmac239 viral load is defined as the average of all viral load timepoints from 8 weeks post-infection until the final available timepoint for a particular RM. ^*b*^Number of CTL populations included for correlation analysis. Criterion for inclusion is the existence of the indicated CTL population at detectable levels above background by pMHCI tetramer staining or CD107a/ICS assay, depending upon which analysis was employed to evaluate the phenotypic parameter of interest (see “Materials and Methods” section). ^*c*^Analyses yielding non-statistically significant *p*-values (*p* > 0.05) are not depicted in table.*

## Discussion

The phenomenon of CTL exhaustion poses a formidable challenge for the development of CTL-based HIV/SIV vaccination and cure strategies. However, recent studies have demonstrated effective retargeting of exhaustion-refractory CMV-specific CTL populations to kill tumor cells, suggesting that this strategy could also be applied to HIV/SIV infection. Here we report the phenotypic and functional characterization of two RhCMV-specific CTL populations that do not become exhausted during chronic SIVmac239 infection of RMs, despite moderate levels of SIVmac239-induced immunosuppression via CD4^+^ T cell depletion. Of the eleven *Mamu-A^∗^02*^+^ RMs included in this study, ten had at least one of the two RhCMV-specific CTL populations in circulation. Furthermore, these CTLs did not change in frequency, phenotype, or effector function profile between pre- and chronic SIVmac239 infection timepoints. When compared with SIVmac239-specific CTLs, RhCMV-specific CTLs generally contained higher frequencies of T_EM2_, granzyme B^+^, and polyfunctional lymphocytes, and lower frequencies of PD-1^+^ and CTLA-4^+^ lymphocytes. Collectively, our findings suggest that CMV-specific CTLs are suitable for retargeting-based HIV/SIV immunotherapeutic strategies.

We observed that RhCMV-specific CTLs were overwhelmingly (>90%) T_EM2_ (CD28^–^ CCR7^–^), both before and during SIVmac239 infection. This is consistent with previous characterizations of HCMV-specific CTLs in humans, which are predominantly of late/terminally differentiated memory phenotypes (CD28^–^ CCR7^–^ CD27^–^ CD45RA^±^) (12, 16, 17). We also found that RhCMV-specific CTLs had higher T_EM2_ frequencies than SIVmac239-specific CTLs during chronic SIVmac239 infection, which is consistent with a previous report comparing HCMV- and HIV-specific CTLs during chronic HIV infection (12). In both humans and RMs, these terminally differentiated effector memory phenotypes are ideal for mounting rapid and efficacious CTL responses (40), and eliciting CTLs with these phenotypes has been the objective of numerous HIV/SIV vaccination strategies (41). Consistent with their effector memory phenotype, RhCMV-specific CTLs expressed high levels of granzyme B at all timepoints analyzed, and granzyme B^+^ frequencies were higher in RhCMV-specific CTLs than in SIVmac239-specific CTLs during chronic SIVmac239 infection. Most strikingly, frequencies of polyfunctional CTLs were significantly higher in RhCMV-specific CTLs than in any of the four SIVmac239-specific CTL populations analyzed during chronic SIVmac239 infection. Importantly, the polyfunctionality of RhCMV-specific CTLs was maintained during chronic SIVmac239 infection. Similarly, Hosie et al. found that HCMV-specific CTLs express high levels of granzyme B and are highly polyfunctional, albeit in a cohort of healthy, HIV-naïve individuals (15). In the context of HIV infection, Petrovas et al. and Trautmann et al. both compared the effector function profiles of HIV- and HCMV-specific CTLs, but neither evaluated CTL polyfunctionality. Both studies noted enhanced TNF-α production by HCMV-specific CTLs, but found no differences in IFN-γ or IL-2 production between HIV- and HCMV-specific CTLs (11, 12). Nevertheless, our results indicate that RhCMV-specific CTLs naturally adopt effector memory phenotypes conducive to rapid execution of multiple effector functions.

We observed relatively high PD-1^+^ frequencies in both RhCMV-specific CTL populations before and during SIVmac239 infection. However, two of the SIVmac239-specific CTL populations studied (Gag CM9 and Nef YY9) exhibited even higher PD-1^+^ frequencies, and significantly higher PD-1 MFIs, in chronic infection. Interestingly, and rather paradoxically, we found that PD-1^+^ frequencies of Gag CM9-specific CTLs were negatively correlated with setpoint SIVmac239 viral loads. Based on conventional understanding of PD-1-mediated CTL exhaustion, one would expect a positive correlation to exist between these parameters, as increased levels of antigen would presumably result in elevated PD-1 expression by antigen-specific CTLs. Indeed, positive correlations between CTL PD-1 expression and HIV viral loads have been previously reported (42). Previous studies have shown that PD-1 expression is significantly higher in HIV-specific CTLs than HCMV-specific CTLs during chronic HIV infection (11, 12). While two of the SIVmac239-specific CTL populations analyzed in this study had higher PD-1^+^ frequencies (Gag CM9 and Nef YY9) than the RhCMV-specific CTL populations, the other two SIVmac239-specific CTL populations (Tat SL8 and Gag GY9) had PD-1^+^ frequencies comparable to those of RhCMV-specific CTLs. Lower PD-1 expression in Tat SL8-specific CTLs can likely be attributed to early viral escape in the Tat SL8 epitope, which is known to occur during the first 4–6 weeks of infection in *Mamu-A^∗^01^+^* RMs (7, 8). The relative PD-1^+^ frequencies of Nef YY9- and Gag GY9-specific CTLs are peculiar, however. Based on their relative susceptibilities to viral escape (29), one would expect their relative PD-1^+^ frequencies to be inverted. However, because the functional avidities of Nef YY9 and Gag GY9 are both high and differ by only a factor of two (the functional avidities of Tat SL8 and Gag CM9 differ by a factor of 74) (8, 29), it remains possible that viral escape in Gag GY9 could occur before Nef YY9 in certain animals. Nevertheless, because PD-1^+^ frequencies among RhCMV-specific CTLs did not change between pre- and chronic SIVmac239 infection timepoints, the relatively high PD-1 expression of RhCMV-specific CTLs cannot be attributed to SIV-induced immune dysfunction. While PD-1 expression by CMV-specific CTLs is unsurprising, due to the chronic nature of CMV infection, the disparity in PD-1 expression between RhCMV- and HCMV-specific CTLs is puzzling.

We also evaluated CTLA-4 expression in RhCMV- and SIVmac239-specific CTLs upon stimulation. In HIV infection, CTLA-4 upregulation is generally observed in HIV-specific CD4^+^ T cells, rather than HIV-specific CD8^+^ CTLs (43, 44). However, numerous studies have shown that CTLA-4 blockade can enhance antiviral or antitumor CD8^+^ CTL responses (45–48). Indeed, CTLA-4 blockade facilitates tumor infiltration by CD8^+^ CTLs in melanoma, the first disease for which CTLA-4 blockade obtained FDA approval (49). The role of CTLA-4 expression by CD8^+^ CTLs in SIV infection remains unclear, with a number of studies showing inconsistent results regarding the effect of CTLA-4 blockade on SIV-specific CD8^+^ CTLs (50–53). All four of the SIVmac239-specific CD8^+^ CTL populations analyzed in the present study expressed CTLA-4 upon stimulation, to varying degrees. Gag CM9-specific CTLs in chronic infection exhibited the highest CTLA-4 expression, with an average of 20.5% of responding CTLs staining positive for CTLA-4. Positive correlations between CTLA-4 expression and setpoint SIVmac239 viral loads were observed for three of the four SIVmac239-specific CTL populations we studied, which is consistent with the principle that CTL exhaustion results from persistent high-level antigenic stimulation. In contrast, very low frequencies (<2.5% on average) of RhCMV-specific CTLs expressed CTLA-4 upon stimulation, both at pre- and chronic SIVmac239 infection timepoints. Analysis of CTLA-4 MFIs yielded similar results, indicating that both RhCMV-specific CTL populations express significantly lower amounts of CTLA-4 than SIVmac239 Gag CM9- and Nef YY9-specific CTLs. Therefore, not only do our findings indicate that RhCMV-specific CTLs are refractory to CTLA-4-mediated exhaustion, but they confirm that SIV-specific CD8^+^ CTLs do indeed express CTLA-4. Nonetheless, the functional significance of CTLA-4 expression by SIV-specific CTLs remains unclear.

Despite revealing important information regarding the phenotypic and functional characteristics of RhCMV-specific CTLs in chronic SIVmac239 infection of RMs, our study had several limitations. Due to the paucity of RhCMV-derived CTL epitopes restricted for MHC I molecules other than Mamu-A^∗^02, we were confined to analyzing Mamu-A^∗^02-restricted, RhCMV-specific CTL populations. Future studies should investigate RhCMV-specific CTL populations of other MHC I restrictions to determine if they are also refractory to SIVmac239-mediated immune dysfunction. Five of our 18 RMs exhibited control of SIVmac239 replication during chronic infection, to varying degrees. Three of these animals were positive for *Mamu-B^∗^08* or *Mamu-B^∗^17*, RM MHC I alleles strongly associated with spontaneous control of SIVmac239 replication (37, 38). While correlation analyses revealed some statistically significant associations between CTL phenotype and chronic-phase SIVmac239 viremia, the biological significance of these findings is unclear. Furthermore, due to the small number of controllers in our study, we were unable to meaningfully compare RhCMV-specific CTL characteristics between controllers and non-controllers. However, due to the much slower progression of immune dysfunction in SIVmac239 controllers, we can infer that RhCMV-specific CTLs would likely remain functional during chronic infection in these animals. Nevertheless, future investigations of RhCMV-specific CTL functionality in SIVmac239-infected RMs should include more comprehensive analyses of CTL characteristics in RMs with genetic predispositions for control of SIVmac239 replication.

Overcoming the challenges of CTL escape and exhaustion in HIV/SIV infection may ultimately require the retargeting of other endogenous CTL populations exhibiting favorable phenotypes and cytolytic effector functions throughout chronic HIV/SIV infection. Our comparative analysis of RhCMV- and SIVmac239-specific CTL populations indicates that RhCMV-specific CTLs maintain their robust effector memory phenotype and polyfunctionality throughout SIVmac239 infection. Exhaustion marker expression by RhCMV-specific CTLs remained constant, even during chronic SIVmac239 infection, suggesting that RhCMV-specific CTLs are refractory to SIV-induced immune dysfunction. Our findings are largely consistent with those of previous studies of HCMV- and HIV-specific CTLs in humans and validate further investigation of retargeting CMV-specific CTLs as a novel immunotherapeutic strategy for HIV/SIV.

## Data Availability Statement

The raw data supporting the conclusions of this article will be made available by the authors, without undue reservation.

## Ethics Statement

The animal study was reviewed and approved by the University of Wisconsin Graduate School Animal Care and Use Committee.

## Author Contributions

BR and DW: conceptualization, data analysis, funding, and writing (original draft). BR: investigation. BR, NP-L, MR, JR, JS, and ER: methodology. JR, JS, and ER: resources. DW: supervision. BR, NP-L, MR, JR, JS, ER, and DW: writing (review and editing). All authors contributed to the article and approved the submitted version.

## Conflict of Interest

The authors declare that the research was conducted in the absence of any commercial or financial relationships that could be construed as a potential conflict of interest.
