# Supplementary material for: Rhesus Cytomegalovirus-Specific CD8+ Cytotoxic T Lymphocytes Do Not Become Functionally Exhausted in Chronic SIVmac239 Infection
Source: Front Immunol. 2020 Aug 12;11:1960. doi: 10.3389/fimmu.2020.01960 (PMC7457070; doi:10.3389/fimmu.2020.01960)
Supplement: Supplementary file 1 [file Data_Sheet_1.PDF]

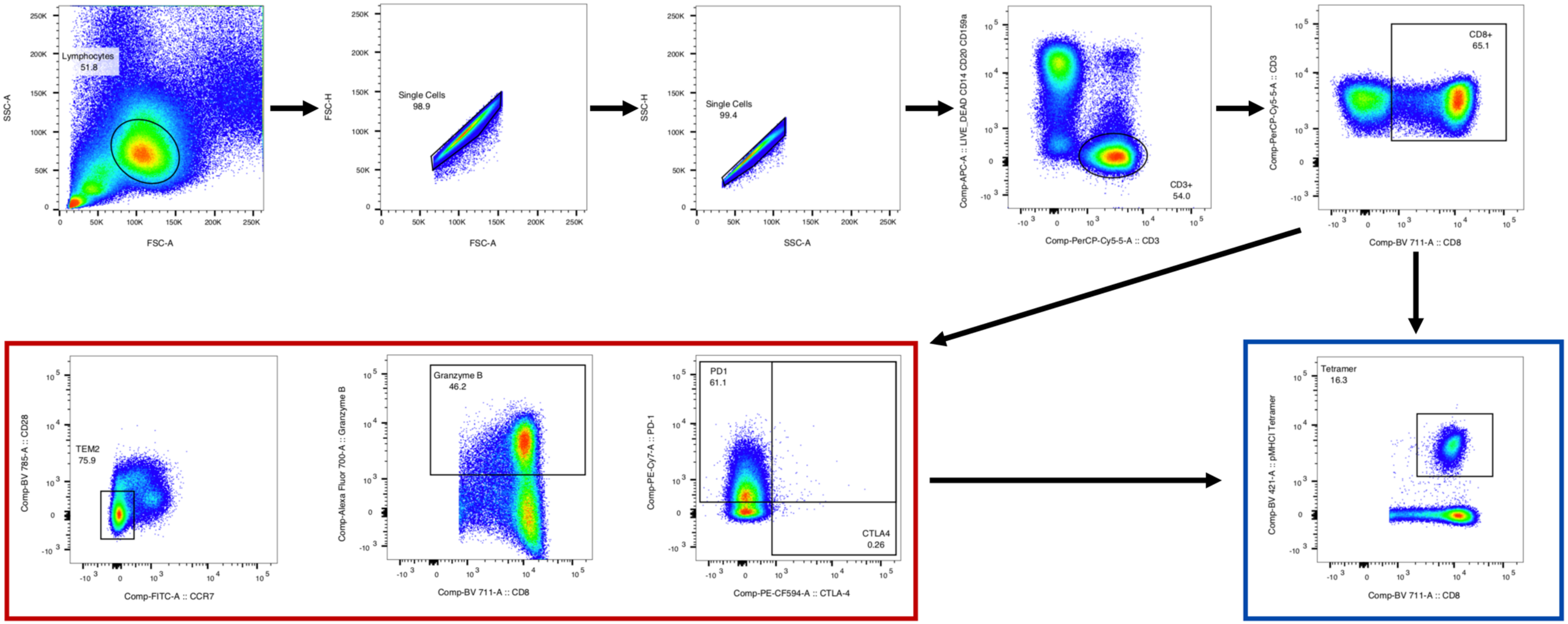

Supplemental Figure 1. Flow cytometry gating strategy for resting PBMC pMHCI tetramer staining. Red boxes show gates which were defined based upon all $CD3^+$ $CD8^+$ T cells in the sample, then applied to the populations of interest boxed in blue ($tetramer^+$ $CD8^+$ T cells).

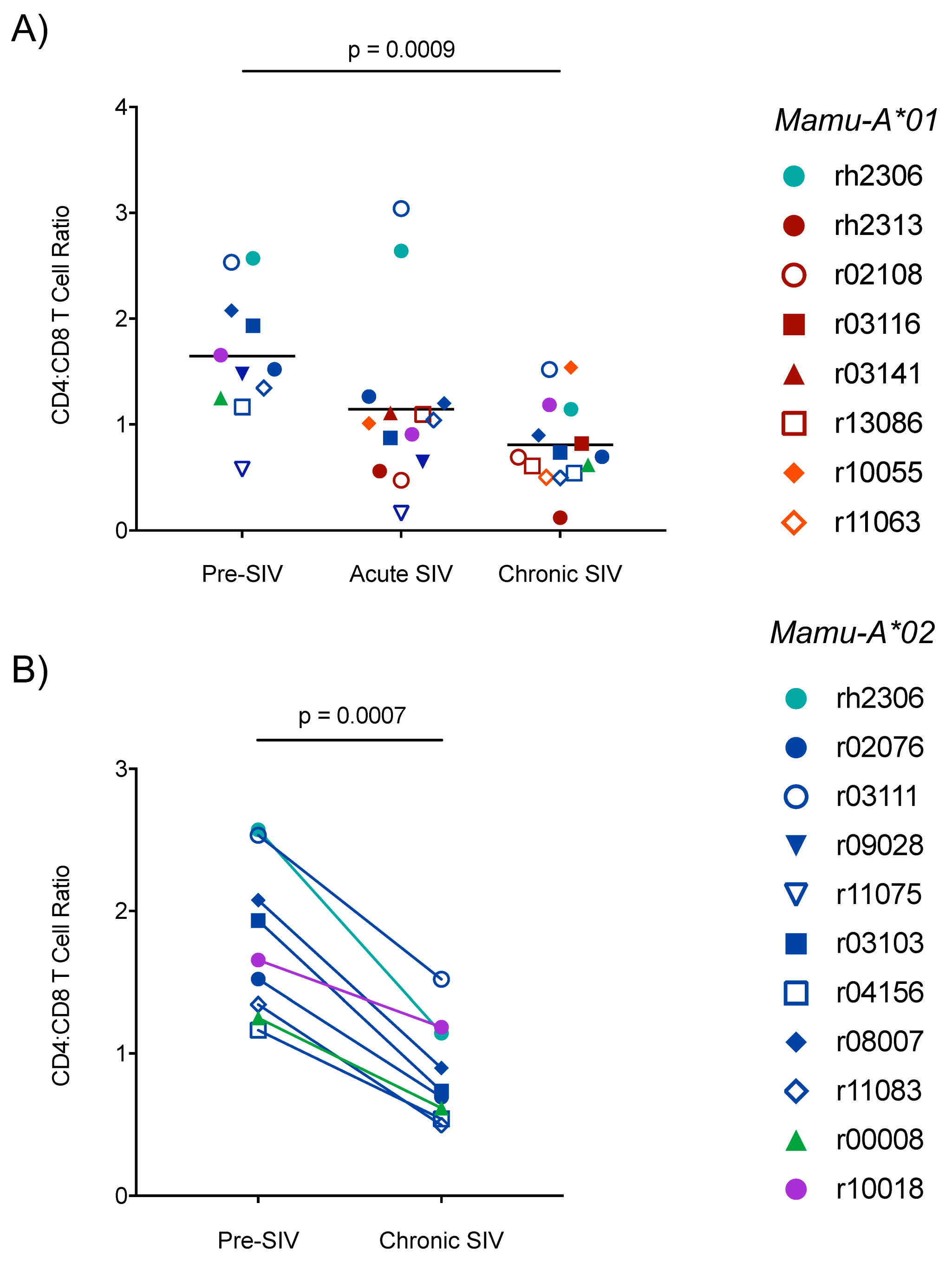

Supplemental Figure 2. CD4:CD8 T cell ratios in SIVmac239-infected RMs. (A) CD4:CD8 T cell ratios for RMs at pre-SIV, acute SIV, and chronic SIV timepoints. (B) CD4:CD8 T cell ratios for all RMs with both pre-SIV and chronic SIV timepoints. CD4:CD8 T cell ratios were determined by analysis of live $CD3^+$ $CD14^-$ $CD20^-$ $CD159a^-$ lymphocytes in resting PBMCs. Ratios were calculated using the following formula: (100 – % of $CD8^+$ cells among live $CD3^+$ $CD14^-$ $CD20^-$ $CD159a^-$ lymphocytes) / (% of $CD8^+$ cells among live $CD3^+$ $CD14^-$ $CD20^-$ $CD159a^-$ lymphocytes). Statistical significance was evaluated using Welch's t-test. In (A), the differences in the CD4:CD8 T cell ratio between the pre-SIV and acute SIV timepoints, and the acute SIV and chronic SIV timepoints, were not statistically significant ($p > 0.05$).

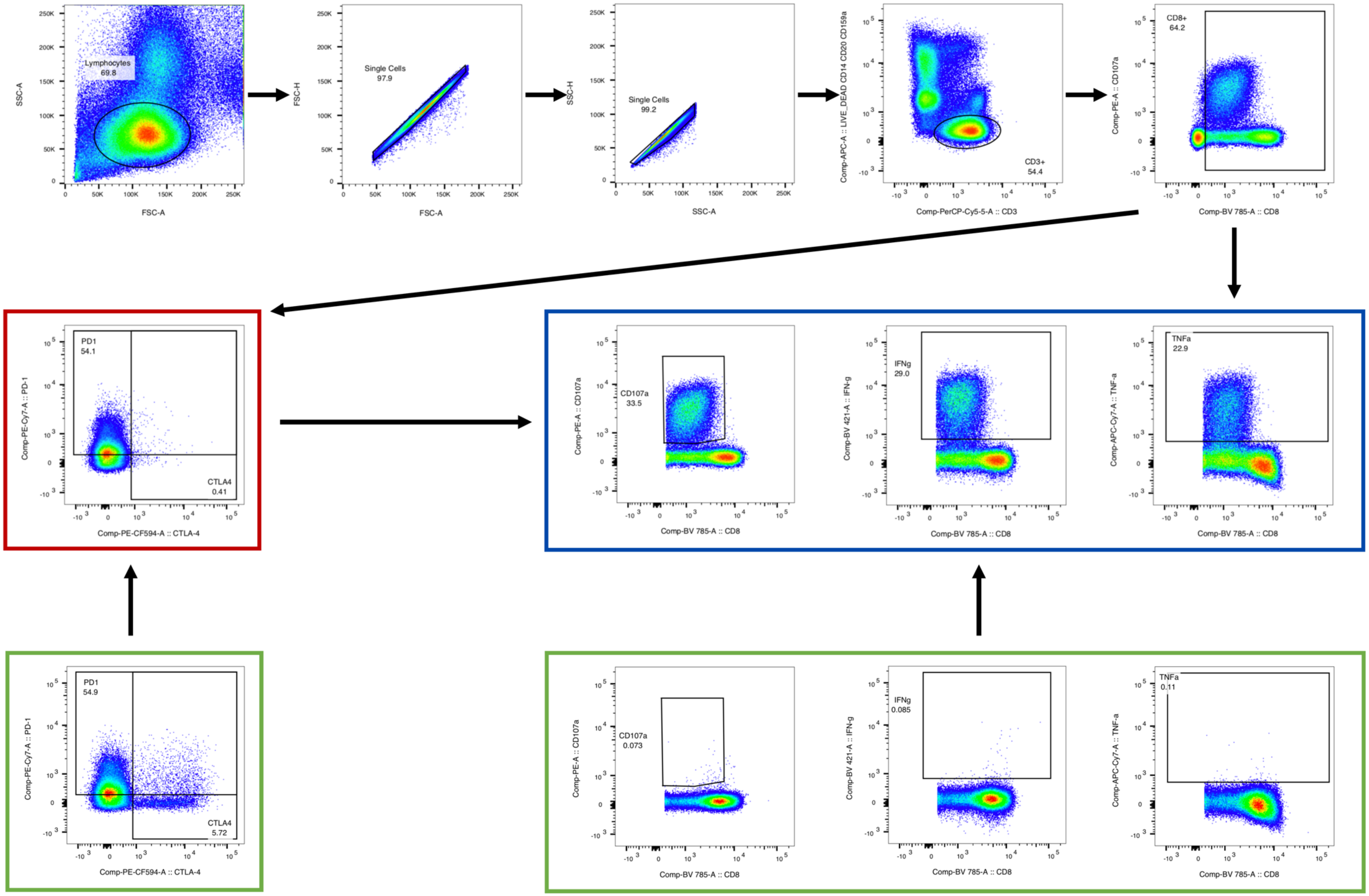

Positive control PBMCs (BD Leukocyte Activation Cocktail)

Negative control (unstimulated) PBMCs

Supplemental Figure 3. Flow cytometry gating strategy for CD107a/ICS assays. PD-1 and CTLA-4 gating was determined using PBMCs from the same animal stimulated with BD Leukocyte Activation Cocktail (positive control for T cell activation; green box at left). Gating for T cell effector function markers (CD107a, IFN-γ, and TNF-α) was determined using unstimulated PBMCs from the same animal (negative control for T cell activation; green box at right). Red box shows gate which was defined based upon all $CD3^+$ $CD8^+$ T cells in the sample, then applied to the populations of interest boxed in blue (responding $CD8^+$ T cells).

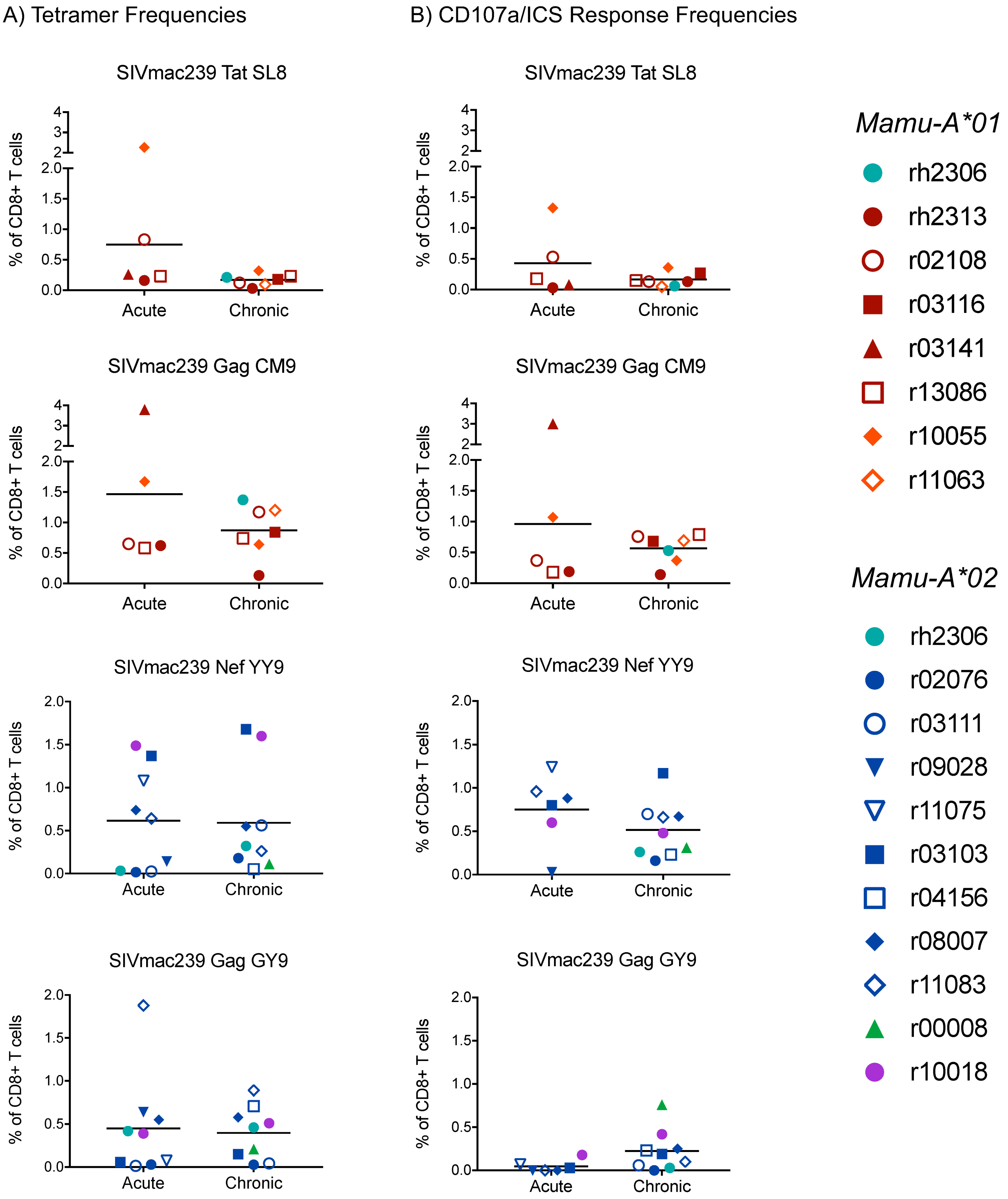

Supplemental Figure 4. Frequencies of SIVmac239-specific CD8$^+$ CTLs during acute and chronic SIVmac239 infection. PBMCs from SIVmac239-infected *Mamu-A*01*$^+$ and *Mamu-A*02*$^+$ animals were stained with (A) the indicated pMHCI tetramers or (B) stimulated with the corresponding minimal optimal peptides in a CD107a degranulation assay with ICS. Graphs depict the frequencies of antigen-specific CD8$^+$ T cells among all CD8$^+$ T cells (defined as live CD3$^+$ CD8$^+$CD14$^-$ CD20$^-$CD159a$^-$ lymphocytes). None of the differences between acute- and chronic-phase CD8$^+$ T cells in panels A and B were statistically significant (all $p > 0.05$, Welch's t-test).

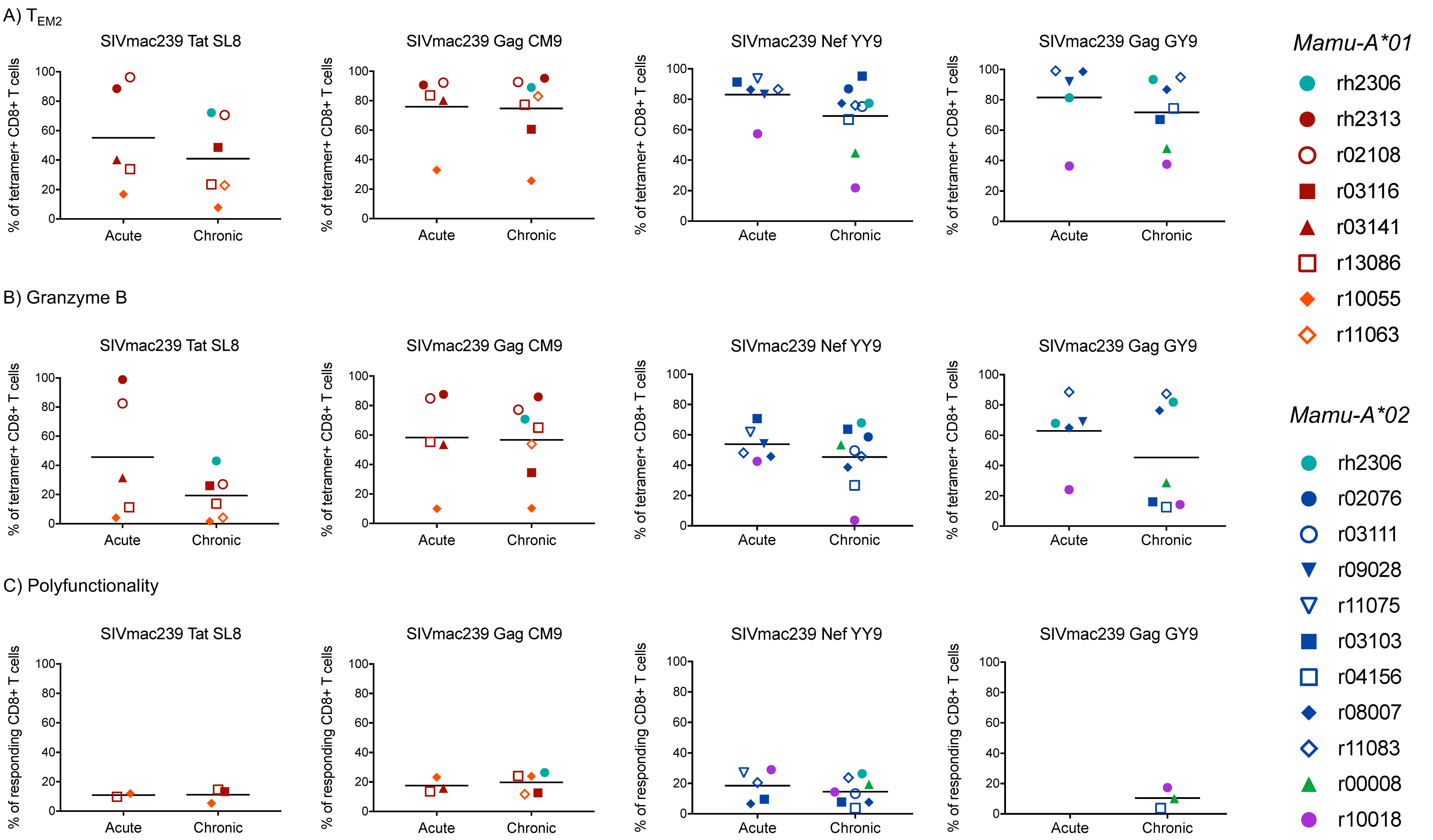

Supplemental Figure 5. Functional characteristics of SIVmac239-specific CD8^{+} T cells during acute and chronic SIVmac239 infection. Graphs depict the frequencies of (A) T$_{EM2}$ (CD28^{-} CCR7^{-}) and (B) granzyme B^{+} cells among tetramer^{+} CD8^{+} T cells (defined as live CD3^{+} CD8^{+} tetramer^{+} CD14^{-} CD20^{-} CD159a^{-} lymphocytes). (C) Frequencies of polyfunctional (CD107a^{+} IFN-γ^{+} TNF-α^{+}) CD8^{+} T cells among CD8^{+} T cells mounting any response (CD107a or IFN-γ or TNF-α) to minimal optimal peptide. In (C), none of the PBMC samples tested at the acute timepoint mounted a detectable response (at least a twofold greater response frequency than the unstimulated negative control) to SIVmac239 Gag GY9. None of the differences between acute and chronic timepoints were statistically significant (all $p > 0.05$ by Welch's t-test).

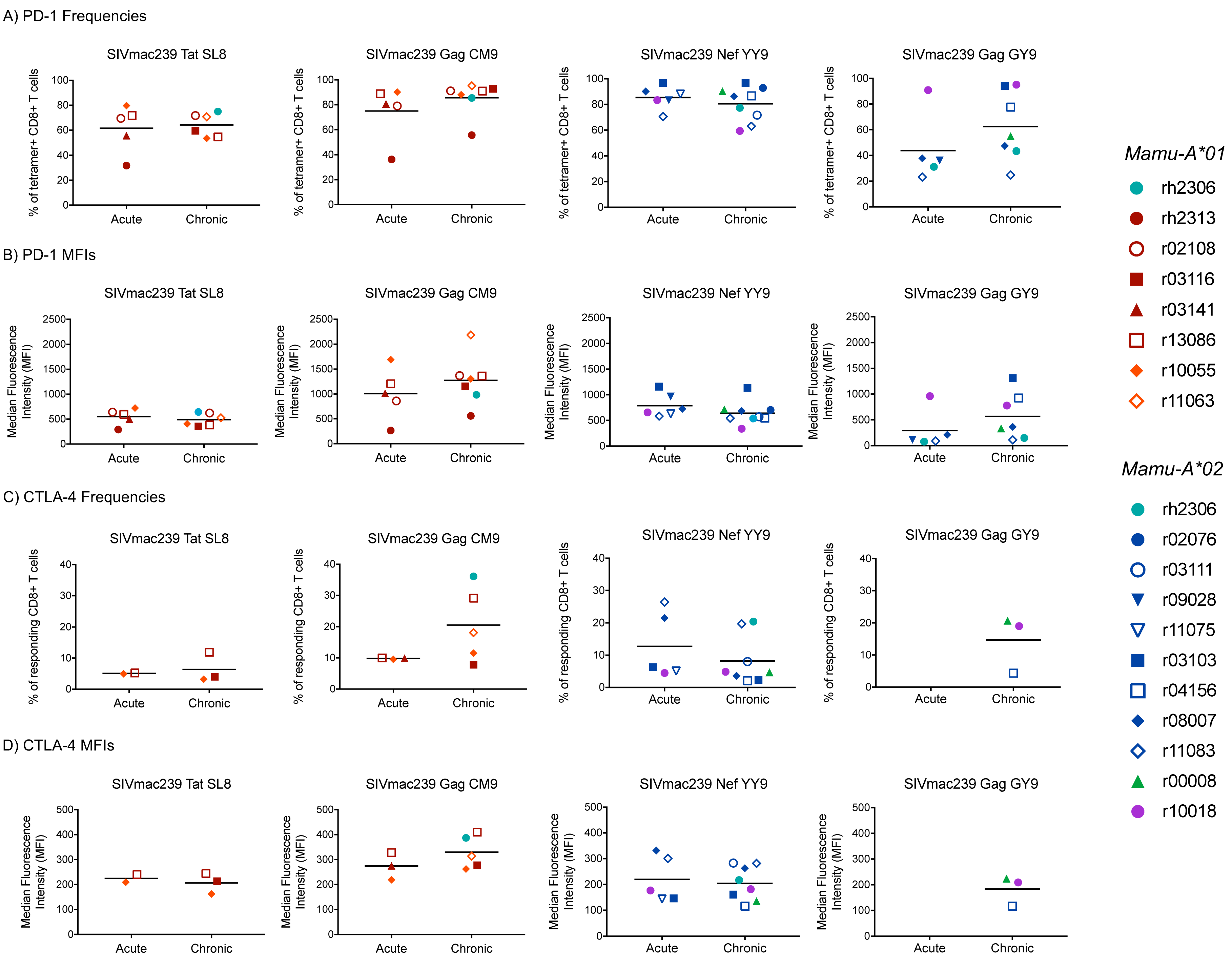
A) PD-1 Frequencies
B) PD-1 MFIs
C) CTLA-4 Frequencies
D) CTLA-4 MFIs
SIVmac239 Tat SL8
SIVmac239 Gag CM9
SIVmac239 Nef YY9
SIVmac239 Gag GY9
% of tetramer+ CD8+ T cells
Median Fluorescence Intensity (MFI)
% of responding CD8+ T cells
Acute
Chronic
Mamu-A*01
rh2306
rh2313
r02108
r03116
r03141
r13086
r10055
r11063
Mamu-A*02
rh2306
r02076
r03111
r09028
r11075
r03103
r04156
r08007
r11083
r00008
r10018

Supplemental Figure 6. Exhaustion marker expression by SIVmac239-specific $CD8^+$ T cells during acute and chronic SIVmac239 infection. (A) Frequencies of PD-1^+ cells among $tetramer^+$ $CD8^+$ T cells and (B) PD-1 median fluorescence intensities (MFIs) for $tetramer^+$ $CD8^+$ T cells (defined as live $CD3^+$ $CD8^+$ $tetramer^+$ $CD14^-$ $CD20^-$ $CD159a^-$ lymphocytes). (C) Frequencies of CTLA-4^+ cells among responding $CD8^+$ T cells and (D) CTLA-4 MFIs for responding $CD8^+$ T cells (defined as live $CD3^+$ $CD8^+$ $tetramer^+$ $CD14^-$ $CD20^-$ $CD159a^-$ lymphocytes mounting any response to minimal optimal peptide ($CD107a^+$ or IFN-γ^+ or TNF-α^+)). In (C) and (D), none of the PBMC samples tested at the acute timepoint mounted a detectable response (at least a twofold greater response frequency than the unstimulated negative control) to SIVmac239 Gag GY9. None of the differences between acute and chronic timepoints were statistically significant (all $p > 0.05$ by Welch's t-test).

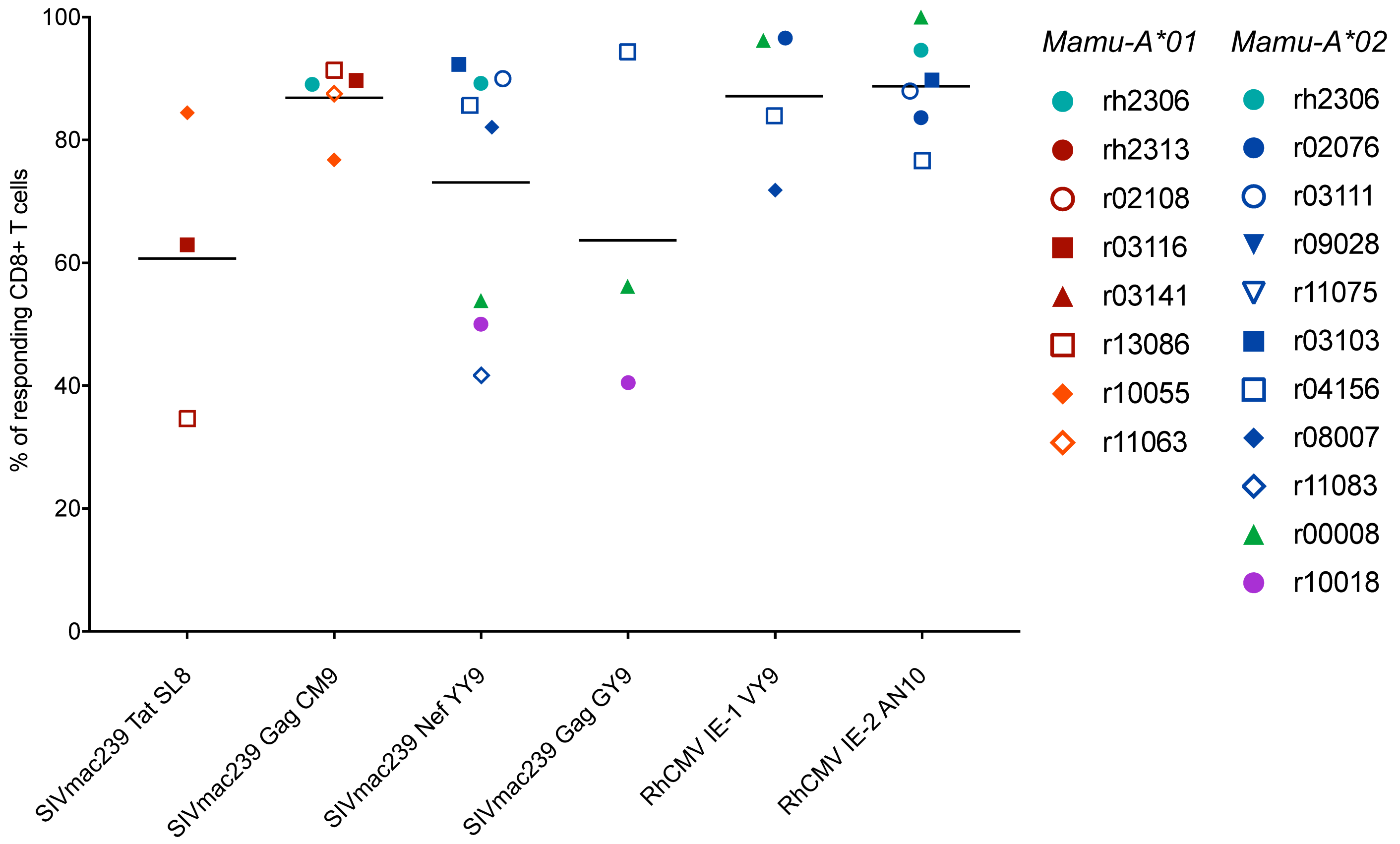

Supplemental Figure 7. Frequencies of degranulating (CD107a$^+$) lymphocytes among RhCMV- and SIVmac239-specific CD8$^+$ T cells during chronic SIVmac239 infection. Graph depicts the frequencies of degranulating (CD107a$^+$) lymphocytes among CD8$^+$ T cells responding to minimal optimal peptide in a CD107a/ICS assay. A responding CD8$^+$ T cell is defined as a live CD3$^+$ CD8$^+$ CD14$^-$ CD20$^-$ CD159a$^-$ lymphocyte staining positive for CD107a or IFN-γ or TNF-α. Statistical significance was evaluated using Welch's t-test. None of the comparisons between RhCMV-specific and/or SIVmac239-specific CD8+ T cell populations yielded statistically significant p-values (not shown on graph, all $p > 0.05$).
